# Multifocal capillary malformations in an older, asymptomatic child with a novel *RASA1* mutation

**DOI:** 10.1111/ced.12696

**Published:** 2015-06-30

**Authors:** S. Whitaker, S. Leech, A. Taylor, M. Splitt, S. Natarajan, N. Rajan

**Affiliations:** ^1^ Department of DermatologyRoyal Victoria InfirmaryNewcastle Upon TyneTyne and WearUK; ^2^ Institute of Genetic MedicineCentre for LifeNewcastle Upon TyneTyne and WearUK; ^3^ Department of DermatologyThe James Cook University HospitalMiddlesbroughUK

## Abstract

Multifocal capillary malformation (CM) is the cardinal feature of patients with *RASA1* mutations. These CMs are ‘red flags’, signalling the possible association with an arteriovenous malformation (AVM) or an arteriovenous fistula (AVF). We report an 8‐year‐old boy who presented with > 20 CMs, who was found to have a novel mutation in the *RASA1* gene. Radiological screening of children with *RASA1* mutations is not standardized, and we elected to carry out baseline magnetic resonance imaging of the brain and spine in our case, which gave normal results. We discuss the recent literature and our approach in the management of such a case.

Multifocal capillary malformation (CM) is the cardinal feature of patients with autosomal dominant inherited *RASA1* mutations, and represents a strong phenotypic marker, present in 97% of mutation carriers.^1^ These CMs are ‘red flags’, signalling the possible association with an arteriovenous malformation (AVM) or arteriovenous fistula (AVF). Clinical management of asymptomatic children with *RASA1* mutations is not standardized, and we discuss the recent literature and our approach in the management of such a case.

## Report

**Figure 1:**
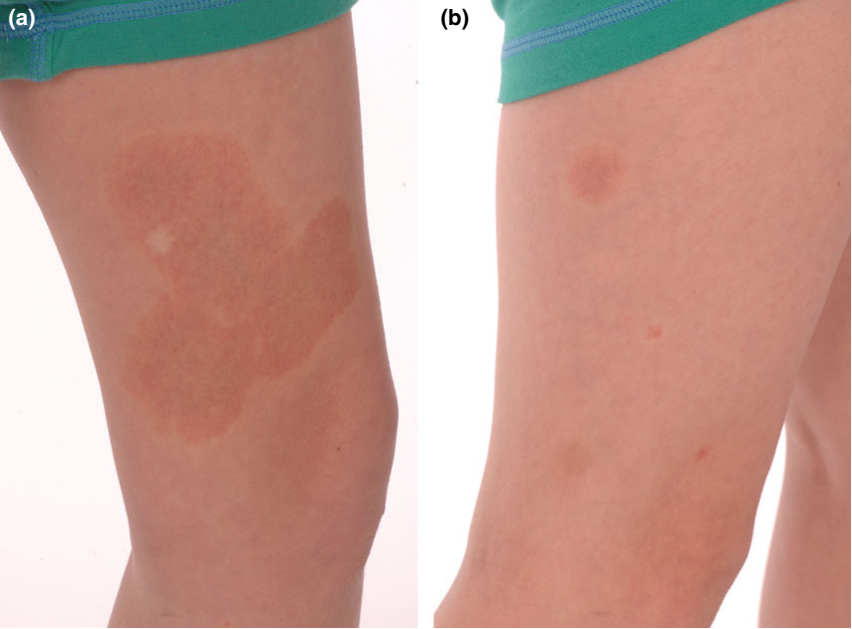
(a) A large capillary malformation with a red–brown appearance and a surrounding pale halo, seen on the medial aspect of the left leg, which had been present since birth. The white lesion within the malformation is a biopsy scar. (b) Smaller similar lesions on the right leg; the smallest of these had appeared in childhood.

An 8‐year‐old boy presented with in excess of 20 CMs, mainly affecting his limbs and neck. These had been present since birth, and new lesions continued developing during childhood. The patient was otherwise well. On physical examination, the lesions were seen to be macular and pink to brown in colour, and some lesions demonstrated a pale halo (Fig. 1a,b). There was a high suspicion of capillary malformation–arteriovenous malformation (CM‐AVM) syndrome. Genetic testing confirmed a novel, heterozygous *RASA1* mutation in exon 18, predicted to create a frameshift and a premature stop codon (c.2467dupG). Magnetic resonance imaging of the brain and spine was performed to exclude the presence of an AVM, and was found to be normal. The proband's mother was examined and found to be unaffected. The father was not available for examination, but was reported to be unaffected.

Familial occurrence of multiple CMs was first described in 1949;^1^ however, it was only in 2002 that molecular characterization of a subset of these patients led to the identification of heterozygous mutations in *RASA1*.^2^ To date, > 100 mutations in this gene have been reported, and no genotype–phenotype correlation is seen.^1^, ^3^ Approximately 30% of *RASA1* mutation carriers have a *de novo* mutation, and the penetrance is high (96.5%).^4^

*RASA1* (ras p21 protein activator 1) encodes the protein p120 RasGAP, an important regulator of vascular cellular differentiation and proliferation. The CMs associated with *RASA1* mutations in humans are different to isolated CMs, and are characterized by a red/brown colour, random distribution and the presence of a whitish peripheral halo in a third of the lesions, suggestive of vascular steal.^5^ The malformations may be present at birth, but usually appear in early childhood, and may increase in number with age.^4^ It has been postulated that a somatic ‘second hit’ genetic mutation is necessary for the development of cutaneous lesions, and this could explain the localized nature, progressive development and multifocality of the lesions. A recently identified loss of the wild‐type allele in CM tissue supports this hypothesis.^1^

In addition to having CMs, 23% of patients with *RASA1* mutations have fast‐flow vascular anomalies: AVM, AVF or Parkes Weber syndrome (PKWS).^2^, ^4^, ^6^ AVMs are rare, fast‐flow anomalies that can cause fatal complications such as bleeding, congestive cardiac failure or neurological deficit. Approximately 10% of these lesions are located in the brain and spinal cord.^7^ The lesions may present at birth but can be asymptomatic for some years. Previously reported intracranial arteriovenous lesions in CM‐AVM in patients with *RASA1* mutations are typically macrofistulas, usually presenting with neurological signs at birth or before 1 year of age. The goal of treatment in cortical AVF in young children, even when asymptomatic, is rapid control of the shunt, because of the high mortality and worse neurocognitive prognosis when managed conservatively. Walcott *et al*. studied a group of *RASA1* mutation carriers who were recognized to be at risk for paediatric pial AVF. These are rare vascular lesions of the CNS, characterized by direct arterial connections to a pial venous channel, which pose a high risk of haemorrhage.^8^ Thiex *et al*. described a previously undocumented association between *RASA1* mutations and fast‐flow anomalies in the spine causing severe sensorimotor defects. In that study, neurological symptoms became apparent as late as at 23 years of age in one patient.^6^

The radiological screening of neonates and young children with a *RASA1* mutation has recently been proposed.^7^ However, reports of older children (> 7 years of age) presenting with *RASA1* mutations and CM without radiologically confirmed intracranial AVM/AVF are limited.^9^, ^10^ Although Revencu *et al*.^4^ reported that most of the intracranial lesions are macrofistulas and cause symptoms in infancy, ascertainment bias cannot be excluded, as their patients may have had imaging arranged as a result of their signs and symptoms.^13^ In addition, prospective studies in *RASA1* mutation carriers to carefully characterize intracranial or spinal AVMs AVFs have not been performed, making estimation of the true prevalence of asymptomatic lesions in childhood difficult in this group.

We suggest that screening using magnetic resonance imaging should be considered in asymptomatic children to identify subclinical high‐flow intracranial and spinal vascular lesions. The existing gap in knowledge as to whether these AVMs and AVFs can progress or develop after such a screening scan remains an obstacle that prevents informed planning of further scans in the absence of symptoms. CMs continue to develop in the skin of these patients in childhood, however, and this emphasizes the importance of educating parents to report new neurological signs early. As further clinical data become available for these older children carrying *RASA1* mutations, intervals for further screening in the absence of new neurological signs can be refined. Although this approach carries the attendant risk of discovering incidental lesions of uncertain significance, it may make it possible to anticipate cerebral and spinal haemorrhages, which could have catastrophic consequences if left untreated.

**Learning points**

- CM is a cardinal feature of patients with *RASA1* mutations.
- An estimated 10% of patients with *RASA1* mutations carry fast‐flow vascular anomalies that affect the brain or spinal cord.
- The majority of these patients present in early childhood with neurological symptoms; however, some can present in the second decade of life.
- We propose baseline imaging of the brain and spinal cord in older, asymptomatic children with *RASA1* mutations to aid early detection and management of fast‐flow lesions.
